# Eccrine Syringofibroadenoma: A Rare Case of Benign Appendageal Tumor

**DOI:** 10.7759/cureus.103336

**Published:** 2026-02-10

**Authors:** Pooja Unnikrishnan, Kirankanth Vudayana, Mohammed Khatija begum, Sai Sriya Chalamalasetty, Pallavi Gullipalli

**Affiliations:** 1 Dermatology, Venereology and Leprosy, Great Eastern Medical School and Hospital, Srikakulam, IND; 2 Dermatology, Venereology and Leprosy, Great Eastern Medical School And Hospital, Srikakulam, IND

**Keywords:** eccrine adnexal tumor, eccrine ductal differentiation, eccrine syringofibroadenoma, rare skin tumor, reactive eccrine proliferation

## Abstract

Eccrine syringofibroadenoma (ESFA) is a rare benign adnexal neoplasm characterized by eccrine ductal differentiation within a fibrovascular stroma and poses significant diagnostic challenges due to its variable clinical presentation. It may occur as a solitary lesion, a reactive proliferation in chronically inflamed or traumatized skin, or in association with hereditary syndromes. We report a case of a 47-year-old male presenting with a two-year history of asymptomatic, slowly enlarging nodules forming an annular plaque over the posterolateral aspect of the right foot. Dermoscopy revealed light-brown pigment globules in a cobblestone pattern with yellowish scales and central crusting. Histopathology demonstrated anastomosing epithelial cords with eccrine ductal differentiation embedded in a mucin-rich fibrovascular stroma, confirming ESFA. The lesion was treated with complete surgical excision, resulting in an excellent outcome. This case highlights the importance of clinicopathological correlation, the supportive role of dermoscopy, and the need for awareness of ESFA in chronic acral lesions.

## Introduction

Eccrine syringofibroadenoma (ESFA) is a rare benign adnexal tumor characterized by anastomosing epithelial cords with eccrine ductal differentiation embedded in a fibrovascular stroma, first described by Mascaró in 1963 [1,2]. It is thought to originate from the acrosyringium or eccrine dermal duct and remains an infrequently encountered diagnosis, contributing to frequent clinical misinterpretation [3,4].

Clinically, ESFA presents with considerable heterogeneity, manifesting as solitary nodules, plaques, or verrucous lesions, most commonly involving acral sites in middle-aged to elderly individuals [5-8]. Due to its nonspecific morphology, it may mimic several benign or inflammatory conditions, necessitating histopathologic confirmation [9]. Dermoscopic features have been described in only a limited number of reports, and no pathognomonic pattern has been established [10].

ESFA is classified into solitary, multiple, nevoid, syndromic, and reactive forms, the latter often associated with chronic inflammation or mechanical trauma [5,11,12]. Although typically benign, rare cases of malignant transformation have been reported, underscoring the importance of accurate diagnosis and management [13-15].

The present case adds to the limited literature by highlighting an acral ESFA with dermoscopic correlation, emphasizing the potential role of chronic friction and the diagnostic value of integrating clinical, dermoscopic, and histopathological findings.

## Case presentation

A 47-year-old male daily-wage laborer presented with a two-year history of an asymptomatic lesion on the right foot. The lesion, initially a small skin-colored papule below the lateral malleolus, gradually enlarged to form an annular plaque with multiple nodular components. The patient denied pain, itching, discharge, ulceration, or any preceding trauma. There was no personal or family history of similar lesions, systemic disease, or neuropathic symptoms. Neurological and vascular examination of the affected foot was normal. Clinical differential diagnoses considered included verruca vulgaris, eccrine poroma, seborrheic keratosis, and squamous cell carcinoma.

**Figure 1:**
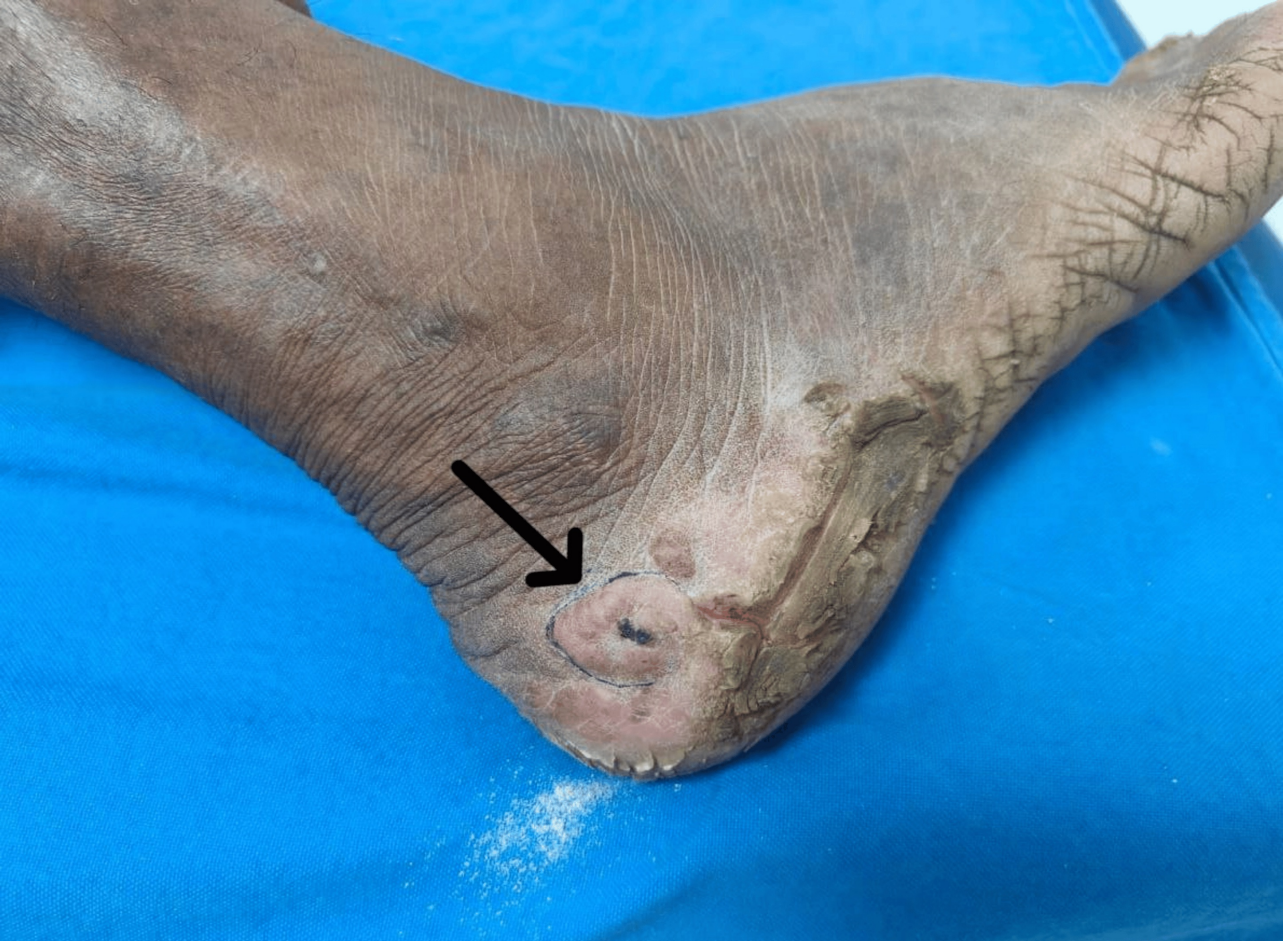
Eccrine syringofibroadenoma Clinical image of the right foot showing multiple pink nodules with xerotic surfaces and central hemorrhagic crusts arranged within an annular plaque below the lateral malleolus.

On examination, a well-defined annular plaque measuring approximately 5 × 3 cm was noted on the posterolateral aspect of the right foot. The plaque was composed of multiple pink nodules that were soft to firm in consistency, with xerotic surfaces and central hemorrhagic crusts. Adjacent to the lesion, there was a thick hyperkeratotic plaque with fissuring. No regional lymphadenopathy was detected (Figure 1).

**Figure 2:**
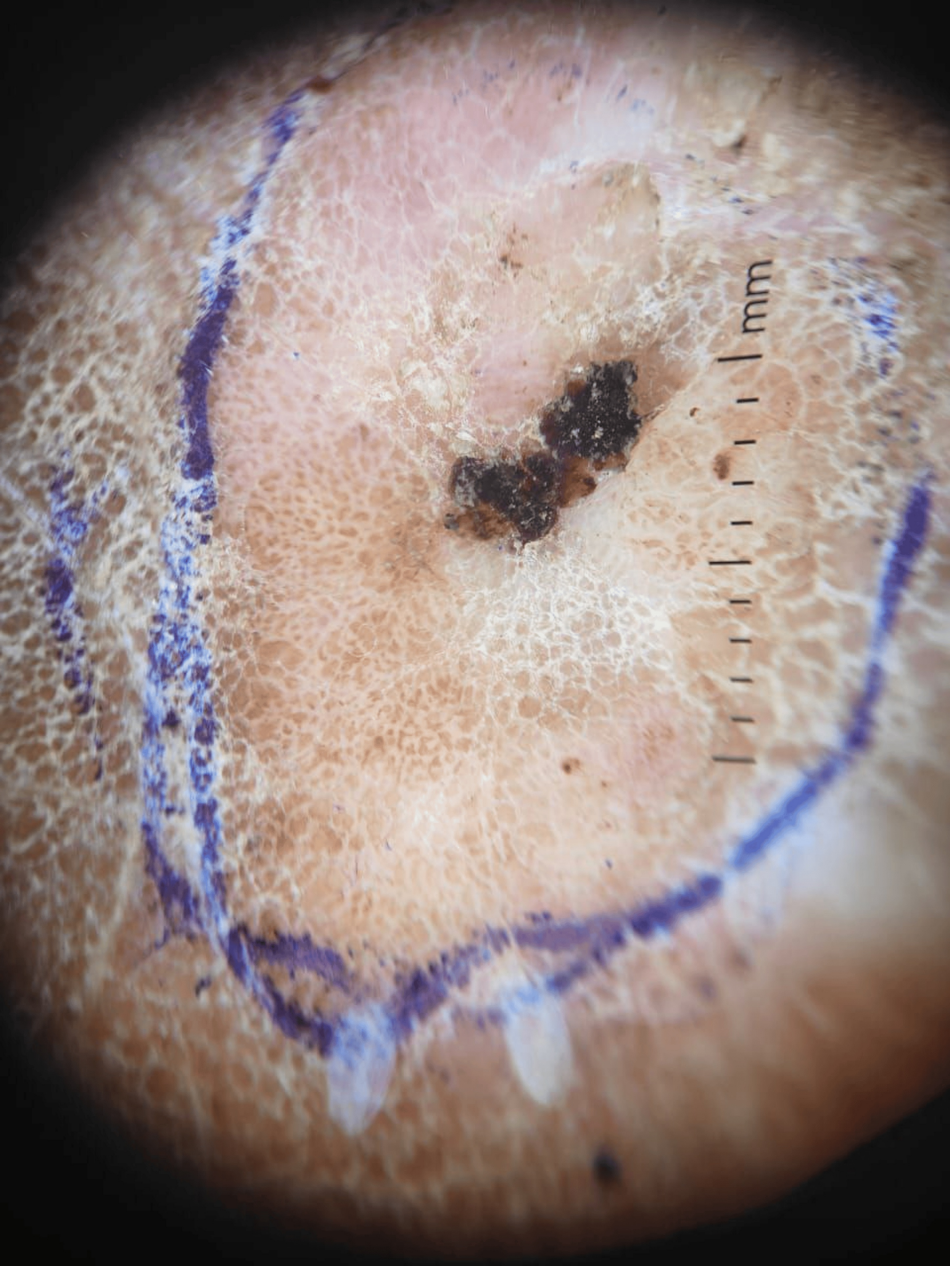
Dermoscopic image demonstrating cobblestone-patterned light brown pigment globules, yellowish scales, and central hemorrhagic crusts over a mildly erythematous background.

Dermoscopy revealed multiple light brown pigment globules arranged in a cobblestone-like pattern on a mildly erythematous background. Yellowish scales and central hemorrhagic crusts were also visible. While the dermoscopic findings suggested an adnexal neoplasm, they were not diagnostic (Figure 2).

**Figure 3:**
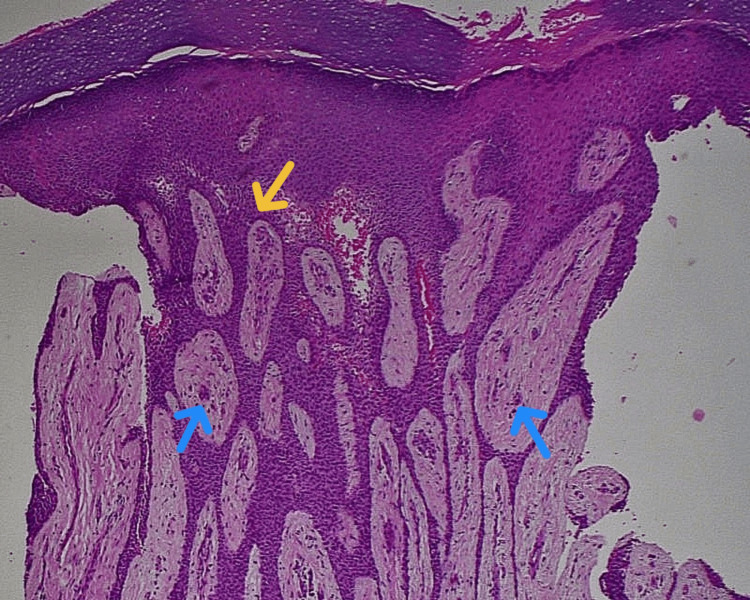
Histopathology (Hematoxylin and eosin stain, ×100) showing anastomosing epithelial strands (yellow arrow) and eccrine ductal structures (blue arrow) within a mucin-rich fibrovascular stroma, consistent with eccrine syringofibroadenoma.

A punch biopsy followed by complete surgical excision was performed to establish a definitive diagnosis and provide therapeutic clearance. Histopathological examination showed marked epidermal hyperplasia with thin anastomosing epithelial strands extending into the dermis. The papillary dermis exhibited mucin deposition and numerous elongated, dilated capillaries. Hyperplastic eccrine ducts were evident throughout the lesion. The deeper dermis showed dense fibroplasia with irregularly arranged thick collagen bundles. No atypia or malignant changes were seen. These findings supported a diagnosis of eccrine syringofibroadenoma (Figure 3).

The lesion was completely excised, and postoperative recovery was uneventful. The patient was followed for six months after excision, with no evidence of recurrence.

## Discussion

Eccrine syringofibroadenoma represents a rare adnexal neoplasm with distinctive histopathologic features enabling differentiation from other cutaneous tumors. The hallmark finding of anastomosing epithelial strands connected to the epidermis embedded in fibrovascular stroma was evident in our case and is consistent with classical descriptions [3,4]. ESFA is considered a tumor of eccrine ductal differentiation, as supported by ultrastructural and immunohistochemical findings demonstrating ductal cell marker expression [3].

Multiple subtypes of ESFA have been described, reflecting its clinical variability. Solitary ESFA, as in this patient, occurs as an isolated lesion and is the most common variant. In contrast, reactive ESFA arises in the context of chronic inflammation or tissue damage, including chronic ulcers, dermatitides, and neuropathic wounds, and may represent a reactive proliferative process rather than a true neoplasm [5,11]. Nevoid and multiple forms are rare and may be associated with genetic syndromes or ectodermal dysplasia [5,12]. The clinical presentation of ESFA is nonspecific; lesions may be hyperkeratotic plaques, nodules, or verrucous growths, often leading to misdiagnosis without histologic evaluation [6,9].

Dermoscopy has a limited role due to the lack of specific features, although some reports describe a mottled vascular or cobblestone appearance [10]. The cobblestone pattern observed on dermoscopy represents clustered, lobulated structures that correspond to epidermal or adnexal proliferations and is not specific to eccrine syringofibroadenoma. Similar patterns may be encountered in benign adnexal tumors such as eccrine poroma and syringoma, as well as in seborrheic keratosis and verruca vulgaris [7,9]. In eccrine syringofibroadenoma, this appearance likely reflects anastomosing epithelial cords with eccrine ductal differentiation embedded in a fibrovascular stroma [1,3]. In patients with skin of color, dermoscopic assessment of adnexal tumors is challenging due to increased background pigmentation and reduced visibility of vascular structures. Pigmented basal cell carcinoma remains an important differential diagnosis and typically demonstrates blue-gray globules, spoke-wheel or maple leaf-like areas, and arborizing vessels [9,12]. The absence of these malignant dermoscopic features, along with clinicopathologic correlation, supported a benign adnexal neoplasm in our case. Therefore, while dermoscopy serves as a useful adjunctive tool, definitive diagnosis of eccrine syringofibroadenoma relies on characteristic histopathological findings [3,7].

Definitive diagnosis depends on histopathologic recognition of branching epithelial cords with ductal differentiation and fibrovascular stroma, often supported by positive immunohistochemical staining for eccrine markers [3,4]. Although benign, ESFA has been associated with malignant transformation, particularly in longstanding or reactive lesions. Solitary eccrine syringofibroadenocarcinoma and carcinoma arising in preexisting ESFA have been documented, illustrating the potential for malignancy and the importance of excision and surveillance [13-15]. Features suggestive of malignancy include cytologic atypia, invasive growth, and loss of typical histologic architecture.

Management of ESFA hinges on the clinical subtype. Solitary tumors are best treated with complete surgical excision, providing resolution and histologic confirmation [7]. Reactive ESFA may resolve with treatment of the underlying trigger, though excision is often performed to exclude malignancy [11]. Follow-up is recommended due to the rare but documented risk of recurrence and malignant change. Our case exemplifies the typical clinical and histopathologic features of solitary ESFA on an acral site. Recognizing ESFA in the differential diagnosis of chronic noduloplaque lesions is critical for appropriate management. Continued reporting and characterization of ESFA subtypes will enhance understanding of its pathogenesis, clinical behavior, and optimal therapeutic approaches.

## Conclusions

Eccrine syringofibroadenoma is an uncommon adnexal neoplasm with varied clinical presentations that often resemble other benign and inflammatory dermatoses, particularly on acral surfaces. This case underscores the importance of maintaining a broad differential diagnosis when evaluating chronic, slowly progressive noduloplaque lesions of the foot. Dermoscopic findings, while supportive, remain nonspecific; thus, definitive diagnosis depends on characteristic histopathologic features demonstrating anastomosing epithelial strands with eccrine ductal differentiation.

Complete surgical excision remains the preferred management approach for solitary ESFA, offering both diagnostic confirmation and curative treatment. Although typically benign, reports of malignant transformation in longstanding lesions highlight the need for histologic assessment and appropriate follow-up. Continued documentation of such cases enhances clinical awareness and contributes to a more comprehensive understanding of ESFA’s behavior, diagnostic challenges, and optimal therapeutic strategies.
